# School Clinics

**Published:** 1921-05-21

**Authors:** 


### SCHOOL CLINICS.

Dr. G. A. Auden, the school medical officer under the Birmingham Education Committee, has raised a point of great importance in his annual report. The outstanding feature of the year's work was the increase in the number of individual children attending school clinics—from 24,795 in 1919 to 29,462 in 1920. This fact, it is true, affords striking evidence of the high estimation in which the clinics are held by parents, but Dr. Auden is fully alive to the danger which it reveals of a perversion of the original preventive-hygienic object of clinics and a reproduction of the worst defects of a crowded outpatient department in a voluntary hospital.

It is not only in Birmingham that the school medical officers are "well-nigh swamped" by the number of children in attendance upon these clinics, and in present circumstances it is often quite impossible to give to individual cases the careful and detached examination which is essential. Local authorities appear to take the view that the curative side of school work is likely to increase, in view of the financial straits of most of the voluntary hospitals and the natural desire of the administrators of these institutions to limit their responsibilities.

But it may be a suggestion worth considering that the treatment of children should be largely transferred from the hospital to the school clinic, except in cases of serious illness or infectious disease. There might be certain practical objections to a proposal of this kind, and it would clearly involve an enormous extension of the school clinic and therefore a heavy initial call upon the public purse. In the long run, however, the saving might be considerable, both in money and from the standpoint of health. The machinery exists and could easily be adapted and developed; the school is a convenient centre at which all children are under close and regular observation; and in all probability parents and children alike would take immediate advantage of this readily available means of guidance and treatment instead of waiting until obvious or serious symptoms had appeared, so that the task of treatment would be made simpler and in many cases more effective. The suggestion is one which, on broad grounds, ought not to be dismissed without adequate examination.

